# Research on Ecoenvironmental Quality Evaluation System Based on Big Data Analysis

**DOI:** 10.1155/2022/5191223

**Published:** 2022-03-07

**Authors:** Pingheng Li

**Affiliations:** Business School, Huanggang Normal University, Huanggang, Hubei 438000, China

## Abstract

Comprehensive and objective evaluation of ecological environment quality is of great significance to regional sustainable development. In this study, Landsat remote sensing images of 1991, 2000, 2004, 2010, 2013, 2018, and 2019 are selected to evaluate the changes of ecological environment quality in the Headwaters of Dongjiangyuan River by using remote sensing ecological index RSEI. The influencing factors of ecological environment change in Dongjiangyuan River are also discussed. The results showed that, from 1991 to 2019, the ecoenvironmental quality of the Dongjiangyuan River showed a good trend of development. Humidity index, greenness index, and dryness index all fluctuated in a small range; the greenness and dryness showed an overall increase. The average temperature in the Headwaters of the Dongjiangyuan River presents a rising trend. This study establishes the evaluation system of ecological environment quality from two dimensions of time and space and gives the change rule of environmental quality quantitatively, which provides the theoretical basis for the ecological environment management of Dongjiangyuan River.

## 1. Introduction

The ecological environment is a compound ecosystem that affects human life and production, which plays an extremely important role. Ecological environment determines the comfort level of human life. Meanwhile, the quality of ecological environment also restricts the development of social economy. In recent years, with the expansion of urban scale, that human beings have damaged the ecological environment increasingly, which results in a sharp reduction of vegetation, soil erosion, reduction in biodiversity, and other negative effects [1, 2]. Therefore, to accurately assess the contradiction between the needs of human development and the ecological environment, to establish an effective quality evaluation system, and to complete the systematic evaluation of the ecological environment for specific regions are hot issues that needed to be solved urgently by all countries in the world [3].

The traditional ecological environment assessment mainly combines survey data for statistical analysis, which has the disadvantages of high cost, being time-consuming and laborious, and subjective interference [4]. The emergence of big data solves this problem. The big data refers to the collection of data that cannot be captured, managed, and processed by conventional software tools within a certain period of time. The high-growth and diversified information asset requires a new processing mode to have stronger decision-making ability, insight, and discovery ability and process optimization ability. The big data is different from the concepts of “massive data” and “superlarge scale data” fundamentally. In addition to considering the data capacity, the big data focuses more on the diversity of data types, the efficiency acquisition speed of data, the variability, authenticity, complexity, and value of data. Pioneering abandons the excessive desire for causality in traditional research methods and pays more attention to correlation [5, 6]. The quality assessment of ecological environmental that is based on the analysis of the large data can do acquisition, processing, analysis, and application of all kinds of spatial data and nonspatial data, such as POI data, track data, and mobile phone signal data as well as the public comments on open source data, by intelligent means, tools, or software in the limited time, compared with the traditional pattern of “data” analysis and evaluation. The statistical analysis that is based on big data can make a comprehensive evaluation of ecological environment quality objectively and quantitatively [7].

The foundation of ecological quality evaluation system based on big data is the way of acquiring big data. With the extensive development of remote sensing technology, it has become an effective way to acquire big data of surface resources by means of satellite images. The data from remote sensing can obtain the distribution of ground facilities, environmental pollution, and other problems quickly and comprehensively, combined with the corresponding analysis means, which can achieve a comprehensive and objective evaluation of ecological environment quality [8, 9]. Based on this, it has great value for research to establish an ecological environment assessment model to systematically evaluate the ecological environment quality of a specific region by using remote sensing to obtain big data [10].

## 2. Related Work

The evaluation of ecological environment quality overseas pays more attention to the practicability and maneuverability of process and result. Paula et al. [11] selected the indexes from the perspective of land suitability, and the index system including natural environment suitability, biological environment suitability, and functional suitability was constructed. The suggestions based on the analysis results for promoting urban sustainable development were put forward. Alateng et al. [12] studied the relationship between urban economic development and environmental quality of 43 countries by using quantitative statistical calculation method and established the well-known environmental Kuznets curve hypothesis. By Richard et al. [13] based on the perspective of ecological security, combining with three subsystems of forest, water area, and grassland, the ecological security evaluation index system was constructed by selecting 11 indexes, and the ecological security of the area near the Colorado River was evaluated scientifically and comprehensively by cluster analysis method. By Valentina et al. [14], using analytic hierarchy process (AHP), an index system with 11 indexes was constructed according to biomolecules and physicochemical elements to evaluate the ecological environment quality in three semiclosed coastal areas. Muhammad et al. [15] constructed the Mediterranean submarine cave ecosystem which uses the theoretical model of structure operation, and the quality index framework (EBQI) was established based on the structural operation theory model, to evaluate the ecological environmental quality of Mediterranean submarine cave and its surrounding environment.

The evaluation of ecological environment quality in China has gone through a long process. From the definition and principle level, it has gradually developed to qualitative and quantitative evaluation research on ecological environment by using mathematical and physical methods. The ecological system evaluation in China is gradually becoming institutionalized and standardized, and the weight treatment of evaluation elements and evaluation content are increasingly enriched [16]. Zhang et al. [17] used the remote sensing images of Tai'an city in two periods; the EI index was graded and evaluated. The results showed that the ecological environment quality was mainly good and distributed in southwest China. Xu et al. [18] selected greenness, humidity, heat, and dryness indexes, and the principal component analysis was used to build an ecological environment quality evaluation system. The proposal and application of the new Remote Sensing Based Ecology Index (RSEI) broadened the research direction of many scholars. Jia et al. [19] established the evaluation system from three aspects: ecological environment level, ecological environment pressure, and ecological environment protection. The index weight was assigned by entropy weight method and the ecological environment quality of Heilongjiang province was dynamically evaluated by comprehensive index method. Huang et al. [20] constructed the fuzzy comprehensive evaluation index system of ecosystem for the region of Yiqiao mining area, Shandong Province, the membership function is obtained from the original data, and the improvement of entropy technology is used to assign weights to indicators, and the ecological health of the mining area is quantitatively studied. Liu et al. [21] chose Bashang Plateau ecological area as the research object, the ecological environment quality, ecological environment quality in mountain ecological area, and ecological environment quality of plain ecological area as the evaluation index system of target layer and selected 6 ecological environmental quality factors that include the ecosystem, the natural resources, the biological diversity, the biological disasters, the food security, and the social ecological system, which constitutes system layer, with the analytic hierarchy process (ahp) to evaluate the index system. Gao et al. [22] used sustainable development theory and ecological economics principle; 12 indexes were selected, from the angle of economy, environment, and society, that constructed the evaluation index system of ecological environment quality of Xi'an city. The analytic hierarchy process and fuzzy comprehensive evaluation are used to evaluate it quantitatively. Zhou et al. [23] selected the factors that lead to ecological fragility. The ecological environment quality of Beipanjiang river basin was analyzed comprehensively and systematically. The law of ecological development in this area was revealed, and there is still a lot of management space in environmental protection. Li et al. [24] took 10 years as the research period; the ecological environment of Chaohu Lake basin was analyzed. The results showed that vegetation coverage and dryness had a greater effect on the ecological environment in this region.

Principal component analysis: Xu et al. [25] calculated and analyzed the data in the index system by principal component analysis model and made a comprehensive evaluation and comparative study on the ecological environmental quality level of all cities in Anhui Province, providing valuable basic data for the development of ecological civilized cities and future environmental planning in Anhui Province. Cheng and Chi [26] in 2011 used the DPSIR framework to build an index system and established an evaluation model based on the nuclear principal component analysis method. Ten representative cities were selected for evaluation. Sun [27] in 2014 constructed the urban ecological environment quality of Nanjing from the two perspectives of environmental pollution and ecological damage. Principal component analysis was chosen as the evaluation method, and the evaluation object was the ecological environment quality of Nanjing in five years. Chun [28] in 2015, based on the basis of a comprehensive evaluation, according to the specific region characteristic and the development of the Xi'an in Shanxi Province, established a comprehensive evaluation index system of eco-city development, using the method of principal component analysis on the comprehensive development of urban ecological quality, and evaluated the quality of each subsystem analysis, according to the result of evaluation data to find out the reason. The evaluation of the coordinated development of each subsystem reflects the current situation of ecological environment quality in Xi'an. Fuzzy comprehensive evaluation method: In the evaluation of environmental quality, Wang [29] in 2017 selected 40 indicators to construct an index system based on the characteristics of the county (city) development from five perspectives of human settlement environment, social equity, industrial structure, ecological construction, and resource utilization and evaluated and analyzed the ecological environment quality of county (city) with fuzzy comprehensive analysis method.

To sum up, a lot of evaluation and analysis work were done by experts and scholars at home and abroad that built ecoenvironmental quality evaluation systems based on different evaluation objects. However, the single evaluation on the space or time of ecological environment was carried out by existing literature, which led to one-sided analysis and conclusion. In this article, with the east of river basin as the research object, the remote sensing and geographic information technology monitoring were used to monitor the ecological and environmental quality of the watershed. The RSEI model was selected to quantitatively study the ecological environment quality of Dongjiangyuan River watershed. The spatial distribution and temporal variation of ecological environment quality in the source basin of Dongjiangyuan River were analyzed. It provides a scientific basis for future environmental management and ecological civilization construction in Dongjiangyuan River basin.

## 3. The Principle and Analysis Method of the Ecoenvironmental Quality Assessment System

In order to evaluate regional ecological quality quickly, Xu proposed the remote sensing ecological index (RSEI). The remote sensing ecological index model is based on remote sensing images to extract greenness, humidity, dryness, and heat index, through principal component analysis; the four factors are integrated to monitor the ecological environment and evaluate the ecological environment quality.

### 3.1. The Principles of RSEI Model

#### 3.1.1. The Humidity Indicators

The humidity index is closely related to the moisture content of vegetation and soil, which is widely used in ecological environment monitoring and evaluation. The range of wet values is between [−1, 1], and the larger the value is, the higher the humidity is. The humidity index can be represented by the wet component in the *k*−*T* variation. Different formulas used by Landsat sensors are as follows:

TM data:

(1) $$\begin{array}{c} \text{WET}=0.0315\rho_{\text{blue}}+0.2021\rho_{\text{green}}+0.3102\rho_{\text{red}}+0.1594\rho_{\text{nir}}-0.6806\rho_{\text{swir}1}-0.6109\rho_{\text{swir}2}. \end{array}$$

OLI data:

(2) $$\begin{array}{c} WET=0.1511\rho_{\text{blue}}+0.1973\rho_{\text{green}}+0.3283\rho_{\text{red}}+0.3407\rho_{\text{nir}}-0.7171\rho_{\text{swir}1}-0.4559\rho_{\text{swir}2}, \end{array}$$

where *ρ*_blue_ is the blue bands, *ρ*_green_ is the green bands, *ρ*_red_ is the red bands, *ρ*_nir_ is the near-infrared band, *ρ*_swir1_ is the reflectivity of shortwave infrared 1, and *ρ*_swir2_ is the reflectivity of shortwave infrared 2.

#### 3.1.2. The Green Degree Index (NDVI)

Normalized Difference Vegetation Index (NDVI) is the most widely used vegetation index, which can effectively reflect the growth status of plants. The range of NDVI value is between [−1, 1], and the larger the value is, the higher the vegetation coverage is. Therefore, normalized vegetation index (NDVI) was used to represent the greenness index. The formula is as follows:

(3) $$\begin{array}{c} \text{NDVI}=\frac{\left(\rho_{\text{nir}}-\rho_{\text{red}}\right)}{\left(\rho_{\text{nir}}+\rho_{\text{red}}\right)}, \end{array}$$

where *ρ*_nir_ and *ρ*_red_ are the near-infrared reflectance and the reflectivity of the red band.

#### 3.1.3. The Dryness Index (NDSI)

The soil drying represents the degree of land exposure and dryness. The continuous desiccation of soil has a serious impact on the ecological environment quality in this region. It is also one of the important factors of ecosystem imbalance. In this paper, the average value of the building index (IBI) and bare soil index (SI) was used to construct the dryness index (NDSI). The NDSI value ranges in [−1, 1], and the higher the value is, the higher the degree of drying is. The formula is as follows:

(4) $$\begin{array}{c} \text{IBI}=\frac{2\rho_{\text{swir}1}/\left(\rho_{\text{swir}1}+\rho_{\text{nir}}\right)-\rho_{\text{nir}}/\left(\rho_{\text{nir}}+\rho_{\text{red}}\right)-\rho_{\text{green}}\left(\rho_{\text{green}}+\rho_{\text{swir}1}\right)}{2\rho_{\text{swir}1}/\left(\rho_{\text{swir}1}+\rho_{\text{nir}}\right)+\rho_{\text{nir}}/\left(\rho_{\text{nir}}+\rho_{\text{red}}\right)+\rho_{\text{green}}\left(\rho_{\text{green}}+\rho_{\text{swir}1}\right)},\\ SI=\frac{\left(\rho_{\text{swir}1}+\rho_{\text{red}}\right)-\left(\rho_{\text{nir}}+\rho_{\text{blue}}\right)}{\left(\rho_{\text{swir}1}+\rho_{\text{red}}\right)+\left(\rho_{\text{nir}}+\rho_{\text{blue}}\right)},\\ \text{NDSI}=\frac{\left(IBI+SI\right)}{2}. \end{array}$$

#### 3.1.4. The Heat Index (LST)

The heat indicators are expressed in terms of surface temperature. The surface temperature is the temperature of the ground that absorbs solar heat radiation, which affects the growth and development of vegetation and has a strong intervention effect on the water cycle. At the same time, it is also one of the factors affecting the evaporation and transpiration of natural water and indirectly affects the change of ecological environment. The larger the VALUE of LST, the higher the temperature. The surface temperature in this paper is obtained by modifying the brightness temperature, and the formula is as follows:

(5) $$\begin{array}{c} L_{\text{TIR}}=\text{gain}\times DN+\text{bias},\\ T=\frac{K_{2}}{ln\left(K_{1}/L_{\text{TIR}}+1\right)}, \end{array}$$

where *L*_TIR_ is radiation calibration of the thermal infrared band; *K*_1_, *K*_2_ are calibration coefficient.

(6) $$\begin{array}{c} \text{LST}=\frac{T}{\left[1+\left(\lambda T/\rho \right)ln\varepsilon \right]}, \end{array}$$

where *T*, *λ*, and *ε* are the brightness temperature, the central wavelength of the thermal infrared band, and the surface emissivity.

TM data: *K*_1_ = 607. 76 W·m^−2^·sr^−1^·*μ*m^−1^, *K*2 = 1260.56 K, gain = 0.055, bias = 1.18243, *λ* = 11.45 *μ*m, *ρ*  = 1.438 ×10^−2^ m·K.

Landsat8 data: *K*_1_ = 774. 89 W·m^−2^·sr^−1^·*μ*m^−1^, *K*2 = 1321.08 K, gain = 3.342, bias = 0.1, *λ* = 10.90 *μ*m, *ρ*  =1.438 × 10^−2^ m·K.

The land surface emissivity is calculated by the NDVI threshold method proposed by Sobrino, and the formula is as follows:

(7) $$\begin{array}{c} \varepsilon =0.004f+0.986,\\ f=\frac{\left(\text{NDVI}-{\text{NDVI}}_{\text{soil}}\right)}{\left({\text{NDVI}}_{\text{veg}}-{\text{NDVI}}_{\text{soil}}\right)}, \end{array}$$

where *f* is the vegetation coverage, NDVI_soil_ is the NDVI value of bare soil or no vegetation-covered area, and NDVI_veg_ is the NDVI value of complete vegetation coverage.

### 3.2. The Principal Component Analysis

#### 3.2.1. The Basic Principles of Principal Component Analysis

The principal component analysis (PCA) was proposed by Karl Pearson, a British mathematician, in 1901, to realize multidimensional data compression. The principal component analysis is a method used in mathematical statistics, mathematical analysis, and mathematical modeling to transform multiple variables into a few variables through mathematical transformation. The principal component analysis is to obtain another set of unrelated variables through matrix transformation, which is the idea of data dimension reduction. The principal component analysis converts complex elements into N principal components when introducing multiple variables, to simplify the problem and achieve scientific and effective results.

#### 3.2.2. The Mathematical Model of Principal Component Analysis

From the basic principle of principal component analysis, it can be known that principal component analysis is an idea of data dimension reduction, which obtains a new set of independent variables with certain correlations through mathematical transformation.

The principal component analysis is described in a mathematical way. For a data set *X* with *n* samples, *X*_1_, *X*_2_ … *X*_*p*_, and *P* variables, the data matrix is

(8) $$\begin{array}{c} X=\left[\begin{array}{c} x_{11}x_{12}\cdots x_{1p}\\ x_{21}x_{22}\cdots x_{2p}\\ \vdots \vdots \vdots \vdots \\ x_{n1}x_{n2}\cdots x_{np} \end{array}\right]\\ =\left[x_{1},x_{2},\ldots x_{p}\right], \end{array}$$

where *x*_*i*_=(*x*_1*i*_, *x*_2*i*_,…*x*_*ni*_)^*T*^,  *i*=1,2,…*p*.

The principal component analysis is to synthesize the original *P* observation *X*_1_, *X*_2_,…*X*_*P*_, with variables to form *P* new variables.

(9) $$\begin{array}{c} \left[\begin{array}{c} F_{1}=a_{11}x_{1}+a_{12}x_{2}+\cdots +a_{1p}x_{p}\\ F_{2}=a_{21}x_{1}+a_{22}x_{2}+\cdots +a_{2p}x_{p}\\ \cdots \\ F_{p}=a_{p1}x_{1}+a_{p2}x_{2}+\cdots +a_{pp}x_{p} \end{array}\right]. \end{array}$$

It can be written as theta:

(10) $$\begin{array}{c} F_{1}=w_{1i}x_{1}+w_{2i}x_{2}+\cdots +w_{pi}x_{p},\;i=1,2\ldots ,p, \end{array}$$

where *x*_*i*_, *F*_*i*_ are all n-dimensional vectors

The above model should satisfy the following three conditions:

1. There is no correlation between *F*_*i*_ and *F*_*j*_ (*i* ≠ *j*, *i*, *j* = 1,2,…,*p*)
2. The variance of *F*_1_ is greater than the variance of *F*_2_, and *F*_2_ is greater than the variance of *F*_3_, and so on
3. *w*_*k*1_^2^+*w*_*k*2_^2^+⋯+*w*_*kp*_^2^=1,  *k*=1,2 …, *p*

If all three conditions are satisfied, the transformation results in new independent variables.

### 3.3. The RSEI Model Calculation Based on Principal Component Analysis

In this study, the principal component analysis was used to integrate greenness, humidity, dryness, and heat, so as to achieve the purpose of expressing information with a single index. Since the dimensions of all indicators are not uniform, normalization of all indicators is required before principal component analysis to make the range of index values [0, 1]. The normalization formula is as follows:

(11) $$\begin{array}{c} N=\frac{I-I_{\min}}{I_{\max}-I_{\min}}, \end{array}$$

where *N* is the normalized pixel value, *I*_*i*_ is the original value of pixel *i*, Imax is the maximum value of pixels, and *I*_min_ is the minimum value of pixels.

After the normalization of humidity, greenness, dryness, and heat indexes, the four indexes are superimposed to synthesize a new layer. Then apply the principal component transformation to the new layer. If ER-Mapper is used for principal component analysis, RSEI0 is obtained by using the formula of the first principal component, and then the remote sensing ecological index is obtained by normalization.

(12) $$\begin{array}{c} {\text{RSEI}}_{0}=1-\left\{\text{PCI}\left[f\left(\text{Wet},\text{NDVI},\text{LST},\text{NDSI}\right)\right]\right\},\\ \text{RSEI}=\frac{\left({\text{RSEI}}_{\text{0}}-{\text{RSEI}}_{0\min}\right)}{\left({\text{RSEI}}_{0\max}-{\text{RSEI}}_{0\min}\right)}, \end{array}$$

where RSEI is the remote sensing ecological index, which ranges in [0, 1]. The higher the RSEI value is, the better the ecological environment quality is. Conversely, the smaller the RSEI value is, the worse the quality of the ecological environment is.

The ENVI software is used for principal component analysis; the larger the first principal component data value obtained, the better the ecological environment quality. Therefore, the PC1 can be normalized directly to generate remote sensing ecological index. The formula is as follows:

(13) $$\begin{array}{c} \text{RSEI}=\frac{\left(PCI-PCI_{\min}\right)}{\left(PCI_{\max}-PCI_{\min}\right)}, \end{array}$$

where the PCI_min_ is the minimum value of the first principal component; the PCI_max_ is the maximum value of the first principal component

## 4. Results and Analysis of Ecological Environment Assessment in Dongjiangyuan River

### 4.1. The Data Source

The original data used in this paper are mainly Landsat series remote sensing images and DEM numerical elevation data, combined with the 1 : 100,000 land-use status map of the study area in 2015 and other basic data, mainly including social and economic data and natural resource data of the study area. For example, soil conditions, vegetation types, per capita GDP, and other data are shown in Tables 1 and 2.

All Landsat series remote sensing data used in this paper came from USGS website. In this paper, three Landsat remote sensing images were selected, namely, September 6, 1998 (Landsat-5 TM), September 1, 2008 (Landsat-5 TM), and August 9, 2017 (Landsat-8 OLI). The remote sensing image data of the three phases were in the same season. The unity of time is fully considered in the selection, and the interpretation results have good space-time contrast, which can meet the needs of this study.

In the process of further index screening, rough set equivalence relation in quantitative analysis method was used to screen the index, and after the index screening, expert consultation method was used to further improve the index system of ecological environment quality in Yunnan Province. By using the combination of interval hesitation fuzzy set and entropy weight method, the index weight could be obtained more accurately by scoring from several experts. Data processing in this paper was completed with the support of SPSS software, and model calculation is realized by EXCEL software.

### 4.2. The Analysis Results of Principal Component

Based on RSEI model, the ecological environment quality model of Dongjiangyuan River watershed was constructed. Firstly, the indexes of humidity, greenness, dryness, and heat were extracted by using remote sensing software, and then the four indexes were normalized and superimposed. The principal component analysis was performed on the superimposed layers, and finally, the ecological environmental quality index of the Dongjiangyuan River source basin was obtained. The method of principal component analysis is adopted to automatically select the characteristic contribution rate of each component, which can effectively avoid the influence of human factors on the evaluation result and make the evaluation result more objective and accurate. Principal component analysis results of indicators in the source basin of the Dongjiangyuan River in each year are shown in Tables 1–4.

As can be seen from the table, the four indicators contributed to PC1, and the contribution rates of characteristic values of PC1 were 77.43, 73.65, 78.91, 71.06, 73.47, 77.47, and 75.05, respectively, with all the contribution rates greater than 70%, indicating that the first principal component integrated most of the information of the four remote sensing ecological factors. The value of humidity index and greenness index is positive sign, indicating that humidity and greenness have positive effect on ecological environment. The value of dryness index and heat index is negative, indicating that dryness and heat have negative effect on ecological environment.

### 4.3. Variation Characteristics of RSEI Index in the Headwaters of Dongjiangyuan River

#### 4.3.1. The Variation Characteristics of Humidity Index

The statistical humidity index of remote sensing data in each period is shown in Table 5.

The wet value is standardized and ranges between [0,1]. In 1991, 2000, 2004, 2010, 2013, 2018, and 2019, the mean values of wet in Dongjiangyuan River were 0.788, 0.766, 0.791, 0.769, 0.840, 0.810, and 0.778, respectively. The humidity index fluctuated in a small range. But the overall trend is up. 

**Figure 1:**
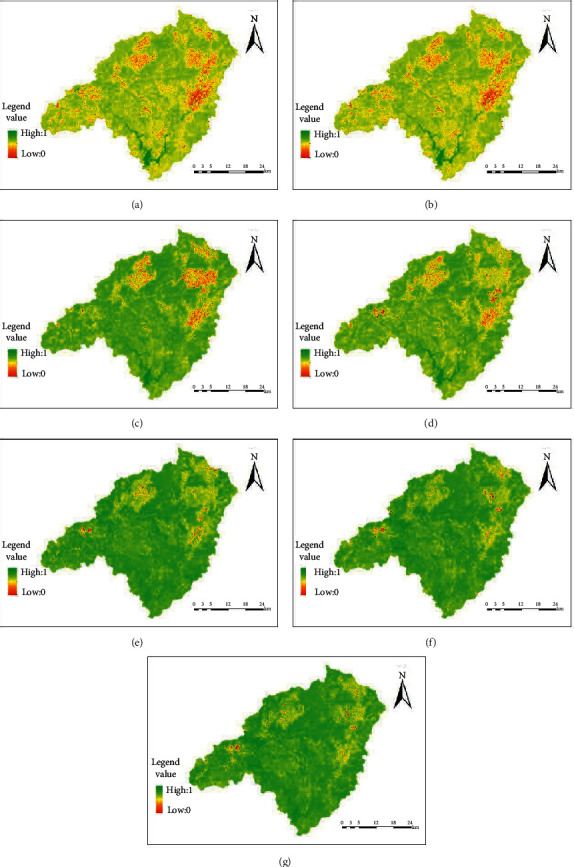
The wet distribution map of remote sensing data in various periods for Dongjiangyuan River: (a) 1991; (b) 2000; (c) 2004; (d) 2010; (e) 2013; (f) 2018; (g) 2019.

The wet distribution maps of the Dongjiangyuan River in each period are shown in Figure 1.

From the spatial distribution map of humidity, it can be seen that the areas with low humidity are mainly distributed in Wenfeng, Changning, Nanqiao, Liuche, Shuiyuan, Zhengang, Kongtian, Hezi, Longtang, Lishi, Tianjiu, Kui Mei Mountain, old town, and township areas. The area covered by vegetation has a higher value of humidity. The distribution of humidity is closely related to human activities.

#### 4.3.2. The Variation Characteristics of Greenness Index

The statistical table of the greenness index of remote sensing data in the source basin of the Dongjiangyuan River in each period is shown in Table 6.

NDVI values are standardized and range between [0, 1]. As can be seen from the table, the mean NDVI values in 1991, 2000, 2004, 2010, 2013, 2018, and 2019 were 0.851, 0.878, 0.768, 0.855, 0.876, 0.884, and 0.887, respectively. The NDVI value increased from 1991 to 2000 but decreased from 2000 to 2004 due to the destruction of ecological vegetation and increased continuously from 2004 to 2019 in the source basin of the Dongjiangyuan River. The NDVI value in the source basin of Dongjiangyuan River showed a trend of first increasing, then decreasing, and then increasing. Because the government issued a series of policies to protect the ecological environment and took vegetation restoration measures, the NDVI value in the source basin of Dongjiangyuan River is increased.

**Figure 2:**
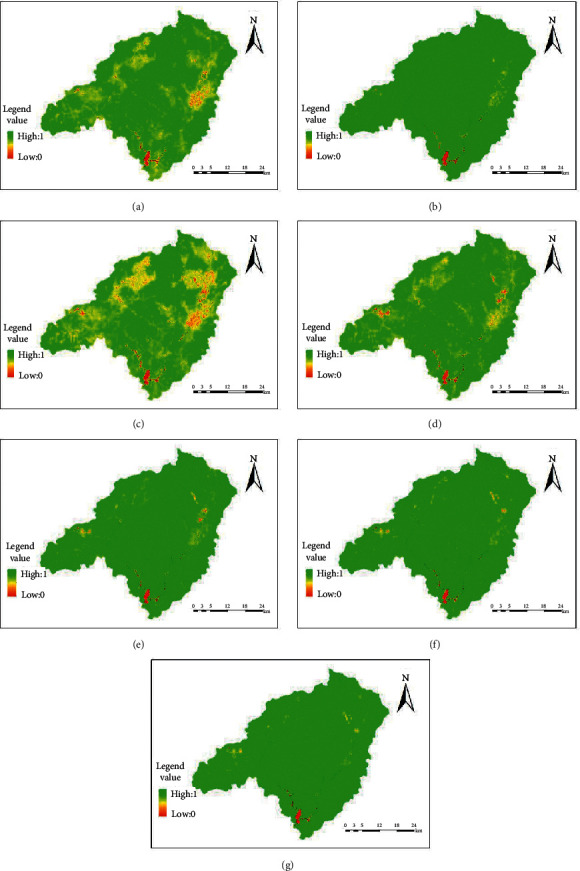
The NDVI distribution map of remote sensing data in various periods for Dongjiangyuan River: (a) 1991; (b) 2000; (c) 2004; (d) 2010; (e) 2013; (f) 2018; (g) 2019.

The distribution diagram of NDVI in the source watershed of the Dongjiangyuan River is shown in Figure 2.

From the analysis of the angle of spatial distribution, Wenfeng, Changning, Nanqiao, Liuche, Shuiyuan, Zhengang, Kongtian, Tachi, Longtang, Lishi, Tianjiu, Kui Mei Mountain, and Laocheng towns show red or yellow, and NDVI is low. The ecological vegetation in these areas has been destroyed by human activities. From 1991 to 2000, the red and yellow areas decreased, indicating that the ecological status of Dongjiangyuan River improved. From 2000 to 2004, the area of red and yellow regions increased, and the ecological environment of some regions deteriorated. From 2004 to 2010, the area of the green zone in Dongjiangyuan River increased and the ecological environment improved. From 2010 to 2013, the scope of green areas in the Dongjiangyuan River further increased, and the ecological environment continued to improve. In 2013, 2018, and 2019, the NDVI of Dongjiangyuan River showed no significant spatial change. From 1991 to 2019, the ecological vegetation status in the Dongjiangyuan River became better.

#### 4.3.3. The Variation Characteristics of Dryness Index

The statistical table of dryness index for remote sensing data in each period in the source basin of the Dongjiangyuan River is shown in Table 7.

NDSI values are standardized and range between [0, 1]. As it can be seen from the table, the mean NDSI values in 1991, 2000, 2004, 2010, 2013, 2018, and 2019 were 0.477, 0.458, 0.623, 0.470, 0.445, 0.434, and 0.432, respectively. The NDSI value decreased from 1991 to 2000, increased from 2000 to 2004, and decreased from 2004 to 2019 in the Dongjiangyuan River. From 1991 to 2019, NDSI values in the Dongjiangyuan River showed a trend of decreasing, then increasing, and decreasing again. From 1991 to 2000, the main reason for the decrease of NDSI was the increase of vegetation area and the decrease of bare land area. From 2000 to 2004, the ecological environment of Dongjiangyuan River was seriously damaged, and the area of bare land increased sharply, which led to the increase of dryness index. The reason why the dryness index of Dongjiangyuan River decreased continuously from 2004 to 2019 is that the government introduced a series of measures to protect the ecological environment, strengthened the management of rare earth mining, and effectively protected the ecological vegetation. 

**Figure 3:**
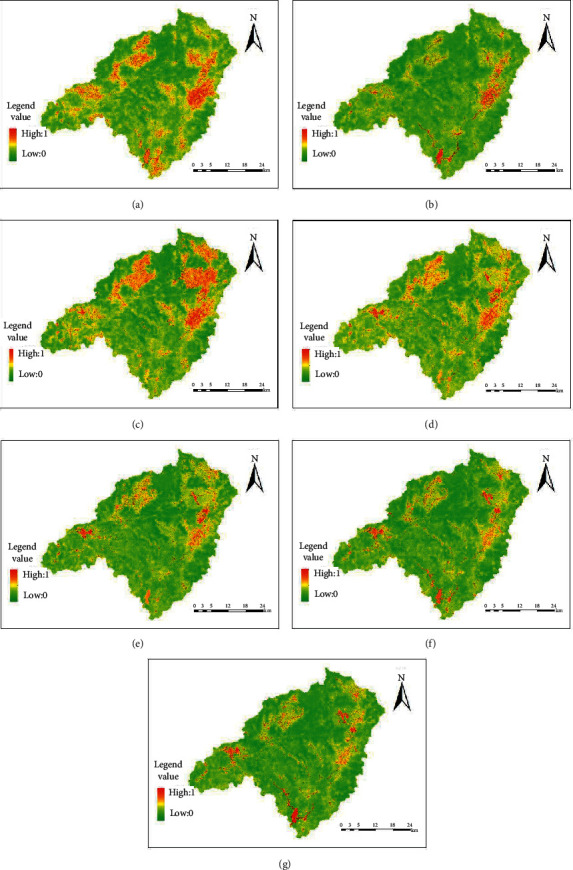
The NDSI distribution map of remote sensing data in various periods for Dongjiangyuan River: (a) 1991; (b) 2000; (c) 2004; (d) 2010; (e) 2013; (f) 2018; (g) 2019.

The NDSI distribution of remote sensing data in the source basin of the Dongjiangyuan River is shown in Figure 3.

In terms of space, Wenfeng, Changning, Nanqiao, Liuche, Shuiyuan, Zhengang, Kongtian, Hezi, Longtang, Lishi, Tianjiu, Kui Mei Mountain, and Laocheng towns show red or yellow, and the large NDSI value indicates a high degree of drying. From 1991 to 2000, the red and yellow areas in the source basin of Dongjiangyuan River decreased, indicating that the degree of drying decreased. From 2000 to 2004, the region of red and yellow regions increased, and the degree of local drying increased. From 2004 to 2010, the area of green area in Dongjiangyuan River is increased, and the degree of drying in the basin is decreased. From 2010 to 2019, the area of the green zone in Dongjiangyuan River kept increasing, and the degree of drying in the basin continued to be improved. On the whole, the degree of drying in the Dongjiangyuan River continued to be improved from 1991 to 2019.

#### 4.3.4. The Variation Characteristics of Heat Index

The statistical table of heat index gained from the remote sensing data in the Dongjiangyuan River in different periods is shown in Table 8.

The mean temperatures in 1991, 2000, 2004, 2010, 2013, 2018, and 2019 were 20.298, 23.247, 21.577, 14.163, 24.922, 23.231, and 26.207, respectively. The temperature has a rising trend as a whole. The average temperature was lower compared with other years in 2010, which is due to the late time of remote sensing images adopted.

**Figure 4:**
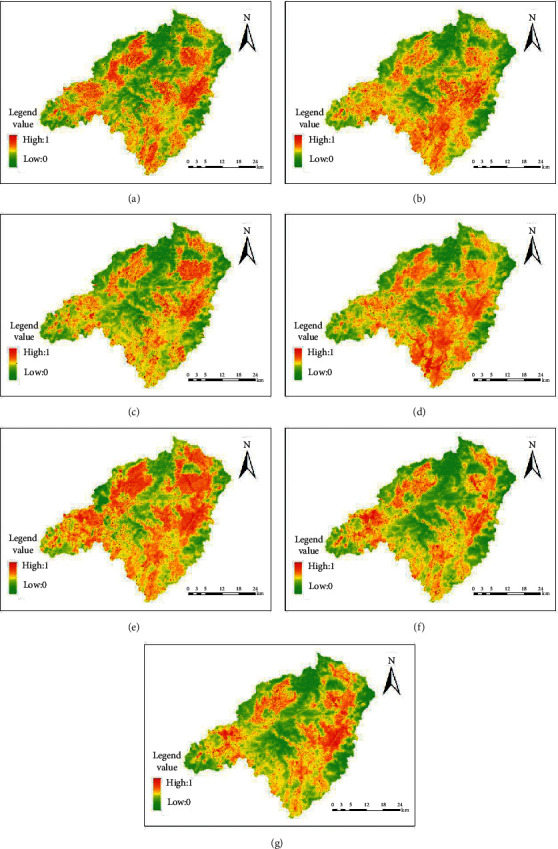
The LST distribution map of remote sensing data in various periods for Dongjiangyuan River: (a) 1991; (b) 2000; (c) 2004; (d) 2010; (e) 2013; (f) 2018; (g) 2019.

The LST distribution map of remote sensing data in various periods of the Dongjiangyuan River is shown in Figure 4.

In terms of space, the red area is mainly distributed in towns and villages around Wenfeng, Changning, Nanqiao, Liuche, Longyan, Chenguang, Changpu, Shuiyuan, Jitan, Zhengang, Kongtian, Keci, Longtang, Lishi, Tianjiu, Kui Mei Mountain, and Laocheng.

### 4.4. The Calculation Results of the Ecoenvironmental Quality Index (RSEI)

Based on Landsat remote sensing image processing, the ecoenvironmental quality index was calculated. The distribution of remote sensing ecological index RSEI in 1991, 2000, 2004, 2010, 2013, 2018, and 2019 in Dongjiangyuan River was 0.628, 0.653, 0.511, 0.540, 0.689, 0.671, and 0.772, respectively. From 1991 to 2019, the ecoenvironmental quality index of Dongjiangyuan River watershed showed a trend of increasing first, decreasing, and then increasing. From 1991 to 2000, vegetation coverage increased and ecological environment quality improved. From 2000 to 2004, the area of bare land increased greatly and the quality of ecological environment deteriorated. From 2004 to 2019, vegetation coverage increased and ecological environment quality improved. On the whole, the ecological environment of Dongjiangyuan River basin is developing in a good direction.

## 5. Conclusion

In this study, seven remote sensing images of 1991, 2000, 2004, 2010, 2013, 2018, and 2019 were selected from the source basin of the Dongjiangyuan River, and the indexes of humidity, greenness, dryness, and heat were extracted by remote sensing processing software. The ecological environment quality evaluation model was built by principal component analysis of four indexes, and the mean values of remote sensing ecological indexes in different years were calculated to analyze the changing trend of ecological environment quality in Dongjiangyuan River. The following conclusions were drawn:

1. From 1991 to 2019, the ecoenvironmental quality of Dongjiangyuan River showed an uptrend, downtrend, and uptrend, and the ecoenvironmental quality was developing in a good direction overall.
2. From 1991 to 2019, the humidity index of the Dongjiangyuan River fluctuated in a small range.
3. From 1991 to 2019, the greenness index in the source basin of the Dongjiangyuan River showed a trend of increasing first, decreasing then, and increasing again.
4. From 1991 to 2019, the dryness index in the Dongjiangyuan River first decreased, then increased, and decreased again; the average temperature in the source basin of the Dongjiangyuan River showed an increasing trend.
5. The change of the land role was an important factor that affected ecoenvironmental quality. The mean values of remote sensing ecological indices of different land types were compared, where the RSEI values of forestland were higher than those of other land types, and the change of the role of land will affect the change of ecological environment quality.

## Figures and Tables

**Table 1:** Principal component analysis results of indexes in 2004 and 2010.

Index	2004				2010			
	PC1	PC2	PC3	PC4	PC1	PC2	PC3	PC4
Wet	0.237	−0.022	0.769	0.593	0.227	0.106	0.832	0.495
NDVI	0.632	0.456	−0.486	0.395	0.534	0.567	−0.467	0.420
NDSI	−0.563	−0.225	−0.373	0.702	−0.451	−0.408	−0,287	0.761
LST	−0.476	0.861	0.179	−0.010	−0.701	0.708	0.083	0.029
Characteristic value	0.017	0.003	0.001	0.000	0.011	0.003	0.001	0.000
Eigenvalue contribution rate	78.91	15.17	5.21	0.71	71.06	21.80	6.44	0.70

**Table 2:** Principal component analysis results of indexes in 2013 and 2018.

Index	2013				2018			
	PC1	PC2	PC3	PC4	PC1	PC2	PC3	PC4
Wet	0.2011	0.031	0.714	0.670	0.183	0.008	0.695	0.696
NDVI	0.607	0.584	−0.460	0.281	0,680	0.492	−0.466	0.282
NDSI	−0.457	−0.252	−0.505	0.687	−0,499	−0.190	−0.528	0.660
LST	−0.618	0.771	0.154	−0.015	−0.506	0.850	0.145	−0.022
Characteristic value	0.012	0.004	0.001	0.000	0.012	0.003	0.001	0.000
Eigenvalue contribution rate	73.47	21.44	4.74	0.35	77.47	17.53	4.64	0.36

**Table 3:** Principal component analysis results of indexes in 1991 and 2000.

Index	1991				2000			
	PC1	PC2	PC3	PC4	PC1	PC2	PC3	PC4
Wet	0.284	0.070	0.864	0.410	0.184	−0.088	0.762	0.615
NDVI	0.479	0.613	−0.426	0.461	0.509	0.369	−0.536	0.564
NDSI	−0.420	−0.404	−0.203	0.787	−0.713	−0.306	−0.308	0.551
LST	−0.716	0.675	0.176	0.009	−0.446	0.873	0.195	0.017
Characteristic value	0.011	0.002	0.001	0.000	0.013	0.003	0.001	0.000
Eigenvalue contribution rate	77.43	16.66	5.23	0.68	73.65	19.16	6.09	1.10

**Table 4:** Principal component analysis results of indexes in 2019.

Index	2019			
	PC1	PC2	PC3	PC4
Wet	0.160	0.012	0.719	0.676
NDVI	0.663	0.537	−0.432	0.293
NDSI	−0.464	−0.221	−0.528	0.676
LST	−0.565	0.814	0.131	−0.020
characteristic value	0.013	0.004	0.001	0.000
Eigenvalue contribution rate	75.05	20.36	4.31	0.28

**Table 5:** Statistical table of humidity index of remote sensing data in each period.

Humidity indicators	Minimum value	Maximum value	Mean value	Standard deviation
1991	0.00	1.00	0.788	0.039
2000	0.00	1.00	0.766	0.035
2004	0.00	1.00	0.791	0.041
2010	0.00	1.00	0.769	0.037
2013	0.00	1.00	0.840	0.031
2018	0.00	1.00	0.810	0.028
2019	0.00	1.00	0.778	0.028

**Table 6:** Statistical table of greenness index of remote sensing data in each period.

Green degree index	Minimum value	Maximum value	Mean value	Standard deviation
1991	0.00	1.00	0.851	0.061
2000	0.00	1.00	0.878	0.066
2004	0.00	1.00	0.768	0.089
2010	0.00	1.00	0.855	0.068
2013	0.00	1.00	0.876	0.077
2018	0.00	1.00	0.884	0.080
2019	0.00	1.00	0.887	0.084

**Table 7:** Statistical table of dryness index of remote sensing data in each period.

Dryness index	Minimum value	Maximum value	Mean value	Standard deviation
1991	0.00	1.00	0.477	0.050
2000	0.00	1.00	0.458	0.0459
2004	0.00	1.00	0.623	0.077
2010	0.00	1.00	0.470	0.052
2013	0.00	1.00	0.445	0.055
2018	0.00	1.00	0.434	0.058
2019	0.00	1.00	0.432	0.058

**Table 8:** Statistical table of heat index of remote sensing data in each period.

Heat index	Minimum value	Maximum value	Mean value	Standard deviation
1991	12.322	34.202	20.298	1.781
2000	13.274	37.514	23.247	1.836
2004	14.218	34.430	21.577	1.678
2010	4.909	25.688	14.163	1.949
2013	15.001	38.442	24.922	1.938
2018	12.198	34.556	23.231	1.599
2019	18.372	41.317	26.207	1.883

## Data Availability

The dataset can be accessed upon request.
